# Supplementary material for: Deletion of wfs1 Impairs Oligodendrocyte Precursor Cells Dorsal Distribution and Myelination Through the wfs1-hmgcs1 Axis in Zebrafish
Source: Biology (Basel). 2026 Aug 16;15(16):1402. doi: 10.3390/biology15161402 (PMC13510151; doi:10.3390/biology15161402)
Supplement: Supplementary file 1 [file biology-15-01402-s001.zip › File S1-Transcriptome sequencing data and differentially expressed genes (DEGs) in wfs1-- zebrafish at 5 dpf/2.KEGG/wfs1KOvsWT/DOWN/src/dre04216.html]

dre04216


- dre:790938

- Down regulated genes

790938(-4.50740250004449)

- dre:100320152

- Down regulated genes

100320152(-1.45074630443396)

- dre:559768

- Down regulated genes

559768(-4.00475710388481)
- dre:558816

- Down regulated genes

558816(-1.68738667729536)
- dre:100126128

- Down regulated genes

100126128(-1.3085831331184)

- dre:559768

- Down regulated genes

559768(-4.00475710388481)
- dre:558816

- Down regulated genes

558816(-1.68738667729536)
- dre:100126128

- Down regulated genes

100126128(-1.3085831331184)

Close
